# Reversine suppresses oral squamous cell carcinoma via cell cycle arrest and concomitantly apoptosis and autophagy

**DOI:** 10.1186/1423-0127-19-9

**Published:** 2012-01-27

**Authors:** Ying-Ray Lee, Wei-Ching Wu, Wen-Tsai Ji, Jeff Yi-Fu Chen, Ya-Ping Cheng, Ming-Ko Chiang, Hau-Ren Chen

**Affiliations:** 1 Department of Medical Research, Chiayi Christian Hospital, Chia-Yi, Taiwan; 2 Department of Nursing, Min-Hwei College of Health Care Management, Tainan, Taiwan; 3 Department of Life Science, Institute of Molecular Biology and Institute of Biomedical Science, College of Science, National Chung Cheng University, Min-Hsiung, Chia-Yi, Taiwan; 4 Department of Biotechnology, Kaohsiung Medical University, Kaohsiung, Taiwan

**Keywords:** Reversine, cell cycle arrest, apoptosis, autophagy, oral squamous cell carcinoma (OSCC)

## Abstract

**Background:**

The effective therapies for oral cancer patients of stage III and IV are generally surgical excision and radiation combined with adjuvant chemotherapy using 5-Fu and Cisplatin. However, the five-year survival rate is still less than 30% in Taiwan. Therefore, evaluation of effective drugs for oral cancer treatment is an important issue. Many studies indicated that aurora kinases (A, B and C) were potential targets for cancer therapies. Reversine was proved to be a novel aurora kinases inhibitor with lower toxicity recently. In this study, the potentiality for reversine as an anticancer agent in oral squamous cell carcinoma (OSCC) was evaluated.

**Methods:**

Effects of reversine on cell growth, cell cycle progress, apoptosis, and autophagy were evaluated mainly by cell counting, flow cytometry, immunoblot, and immunofluorescence.

**Results:**

The results demonstrated that reversine significantly suppressed the proliferation of two OSCC cell lines (OC2 and OCSL) and markedly rendered cell cycle arrest at G2/M stage. Reversine also induced cell death via both caspase-dependent and -independent apoptosis. In addition, reversine could inhibit Akt/mTORC1 signaling pathway, accounting for its ability to induce autophagy.

**Conclusions:**

Taken together, reversine suppresses growth of OSCC via multiple mechanisms, which may be a unique advantage for developing novel therapeutic regimens for treatment of oral cancer in the future.

## Background

Oral cancer is listed as the sixth common tumor worldwide [1]. In Taiwan, oral cancer is even the fourth leading cause of cancer death for males [2]. Oral squamous cell carcinoma (OSCC) is the most common neoplasia and is found frequently in oral cavity such as cheek, gum, and tongue [3]. Although cigarette and alcohol are considered as two major risk factors of oral carcinogenesis [4], occurrence of oral cancer was proved to be tightly associated with betel quid chewing in Taiwan and in south-east Asia [4,5]. So far, surgery and radiation treatments in combination with chemicals like 5-Fu or Cisplatin are the major therapeutic strategies for oral cancers [6,7]. However, surgery and radiation treatments inevitably cause negative impacts on patients' appearance and oral functions like chewing and speaking. In spite of 5-Fu or Cisplatin adjuvant treatments, 5 years survival rate of oral cancer patients is only 30% [6]. A more efficient and safer anticancer drug may be helpful to minimize the surgery area or to delay disease progress.

Aurora kinase, which includes A, B and C members in mammals, is belonged to serine/threonine kinase. Aurora kinase A and B were demonstrated to function at mitosis. Like some cell cycle regulators, expression of aurora kinase A and B oscillates during cell division [8,9]. Aurora kinase A controls the entrance into mitosis by regulating cyclin B/CDK1 [10]. Aurora kinase B phosphorylates Ser10 on Histone H3 to regulate chromosome condensation and interacts with INCENP, survivin, and borealin to form chromosomal passenger complex for chromosome arrangement during cytokinesis [11-14]. Aurora kinase C is mainly expressed in testis and is involved in spermatogenesis [15,16]. Several studies had implicated the relationship between aurora kinases and carcinogenesis [17]. Overexpression of aurora kinase A produces several centrosomes in fibroblast, resulting in aneuploidy [11]. Both aurora kinase A (also named as STK15) and B had been suggested to be correlated with oral cancer [18,19]. Despite its major expression site in testis, aurora kinase C appears occasionally in some cancer tissues [20].

Currently, aurora kinases inhibitors VX680 and PHA-730358 are clinically tested [21,22]. In Myc-overexpressed cells, treatment of VX680 was reported to induce apoptosis or the subsequent autophagy-mediated death in residual cells [23]. Autophagy is a mechanism by which cells enhance metabolism of damaged organelles or recycle dispensable materials to survive harsh conditions like starvation. In the initiation of autophagy, LC3 (type I) could be lipidated and became active form (type II), which would interact with cellular lipid to facilitate aggregation of autophagosome [24]. Therefore, VX680 treatment induces both apoptosis and autophagy, leading to increase the chance of oncolysis. Based on the fact that VX680 successfully interferes with growth of various malignant cell lines obtained from different tissues [25], aurora kinases become valuable targets for cancer therapies. Therefore, it is important to identify effective inhibitors for aurora kinases and understand the mechanisms for the inhibitory effects.

Reversine (2-(4-morpholinoanilino)-6-cyclohexylaminopurine) was found originally to promote cell dedifferentiation [26,27]. Recently, aurora kinases were proved to be the targets of reversine [28]. Compared with VX680, reversine is less toxic to cells from healthy donors but is efficient to reduce cell colony formation from acute myeloid leukemia (AML) patients. Besides, reversine was also proved to block proliferation or to induce programmed cell death in different malignant cell lines such as HCT-116 [29]. *In vivo*, reversine restricts tumor growth from xenograft models experiment [29,30]. These data increase the possibility that reversine may be a potential candidate for treating oral cancers. In this study, we investigate the mechanisms behind the suppressive effects of reversine on OSCC cells and conclude that reversine is a broad-spectrum agent involved in cell cycle arrest, apoptosis, caspase-independent cell death and autophagy.

## Materials and methods

### Cell culture and Transfection

Two OSCC cell lines (OCSL and OC2), which were derived from two males with habits of drinking, smoking, and betel quid chewing in Taiwan, were maintained in RPMI1640 medium supplemented with 10% fetal bovine serum (FBS) and 1% penicillin/streptomycin. Cells were cultured at 37°C supplied with 5% CO_2_. About 1.5 × 10^5 ^cells were seeded in 6-wells plates. Then, cells were transfected with Lipofectamine 2000 (Invitrogen) according to manufacturer's instruction.

### Reagents and antibodies

Reversine was purchased from Cayman. Dimethyl sulfoxide (DMSO) and 3-methyladenine (3-MA) were from Sigma. Trypan blue was from BioWest. Z-VAD-fmk and wortmannin was from Merck. Antibodies against caspase 3, 8, 9, phospho-aurora A/B/C, Cdc2, mTOR, phospho-mTOR, phospho-p70S6K, and rapamycin were from Cell Signaling. Antibodies against AIF, Bcl-xL and Bid were from Epitomics. Antibodies of total and phosphor-Akt were from Santa Cruz. Antibody against LC3 was from Abgent. Other antibodies were all from GeneTex.

### Cells viability analysis

1 × 10^5 ^cells were seeded into 6-wells plates. After chemicals treatment for indicated times, cells were collected by trpsinization, centrifuged, resuspended in 500 μl PBS and stained with 0.5% trypan blue. The unstained cells were quantified using a counting chamber.

### Cell cycle analysis

1 × 10^5 ^cells were seeded in 6-wells plates and serum starved after attachment. After starvation, cells were treated with chemicals, harvested, washed once with 3 ml PBS, centrifuged, resuspended in 1 ml PBS, and finally fixed using 1 ml 100% methanol. The fixed cells were air tightly stored in a 4°C. Before analysis, cells were washed once with 3 ml PBS, resuspended in 400 μl PBS, transferred into a 15 ml tube containing 78 μl 1× PBS, 2.5 μl RNase (10 mg/ml) and 20 μl propidium iodide (PI, 1 mg/ml). After incubation in dark for 30 minutes, treated cells were analyzed by BD CANTO II flow cytometer.

### Cell lysates preparation and Western blot

Cells were lysed with M-PER (mammalian protein extraction reagent, Thermo) containing 0.1% protease inhibitor cocktail. Mixture was vortexed and incubated on ice for 5 minutes. After centrifugation at 14,000 g for 15 minutes at 4°C, protein concentration in the supernatant part was quantified using BCA protein assay kit (Thermo). Proper amount of proteins was mixed with 5× Laemmli sample buffer and boiled for 10 minutes. Samples were run on SDS-PAGE gels and transferred to PVDF membranes. Specific targets were detected using proper antibodies, followed by the secondary antibody conjugated with horseradish peroxidase (HRP). After incubation with Immobilon Western Chemiluminescent HRP substrate (Millipore), the results were detected using BioSpectrum Imaging System (UVP).

### Dual staing of annexin V and propidium iodide (PI)

1 × 10^6 ^cells were seeded into 10 cm plates, treated with indicated chemicals, collected by centrifugation. The pellet cells were stained using Annexin V-FITC detection kit (Strong Biotech) and analyzed by flow cytometer.

### DNA fragmentation analysis

The preparation of fragmented DNA was according the method described [31].

### Immunofluorescence

Cultured cells were washed twice with PBS and fixed in ice-cold 4% formaldehyde/PBS for 20 minutes. After washing three times with PBS again, cells were stained with anti-AIF antibodies at 4°C with gentle swirling overnight. Then, cells were incubated with second antibodies conjugated with Alexa 488 at room temperature. After washing, localization of AIF was observed using a fluorescence microscope.

### Confocal microscope

1.5 × 10^5 ^cells were seeded in 6-wells plates preloaded with sterilized glass cover slips. After transfection and/or chemicals treatment, cells on cover slips were washed twice with PBS and fixed with 4% paraformaldehyde for 30 minutes at room temperature. After washing three times with PBS, cover glasses were carefully mounted onto microscope glasses containing a drop of ProLong Gold antifade reagent with DAPI (Invitrogen). Finally, the slides were sealed and analyzed using Olympus confocal microscope.

## Results

### Reversine suppresses the growth of OSCC cells

**Figure 1:**
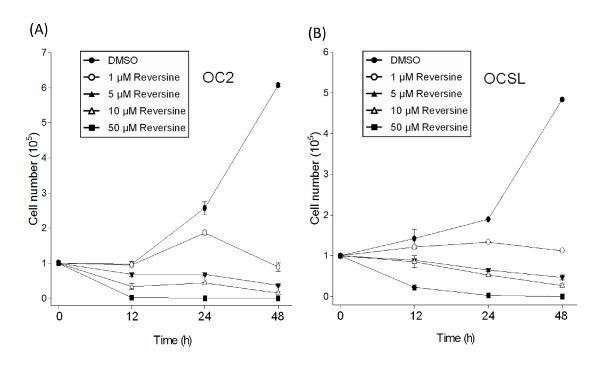
**Reversine suppresses OSCC cell growth**. OSCC cells OC2 (A) and OCSL (B) were treated with DMSO or various concentrations of reversine (1 to 50 μM). Cells were harvested, stained by trypan blue and calculated at the 12, 24 and 48 h. Data were expressed as means ± SD of three independent experiments, each of which was done in triplicate.

To evaluate the potential effect of reversine on suppressing OSCC cell growth, two cell lines OCSL and OC2 cells established from the local patients were examined [32]. The endogenous levels of phosphorylated aurora kinases were detectable, implying the potential application of reversine for suppressing the growth of oral cancer cells (Additional file 1A). To prove that aurora kinase were inhibited in these two cell lines, Serine 10 phosphorylation of histone H3, which was proved to be a direct downstream target of aurora kinases, were evaluated [29,33]. Indeed, reversine obviously inhibited the Serine 10 phosphorylation of histone H3. This result suggested that reversine strongly inhibited aurora kinases in these two OSCC cells (Additional file 1B). Therefore, the effects of reversine on proliferation of OC2 and OCSL were checked. As shown in Figure 1, 1 μM reversine was enough to suppress the growth of both cells. Higher doses of reversine further reduced cell numbers as early as 12 hours after treatment, indicating that reversine possessed the effective inhibition ability against the growth of OSCC cells.

### Reversine interferes with the progress of cell cycle

**Figure 2:**
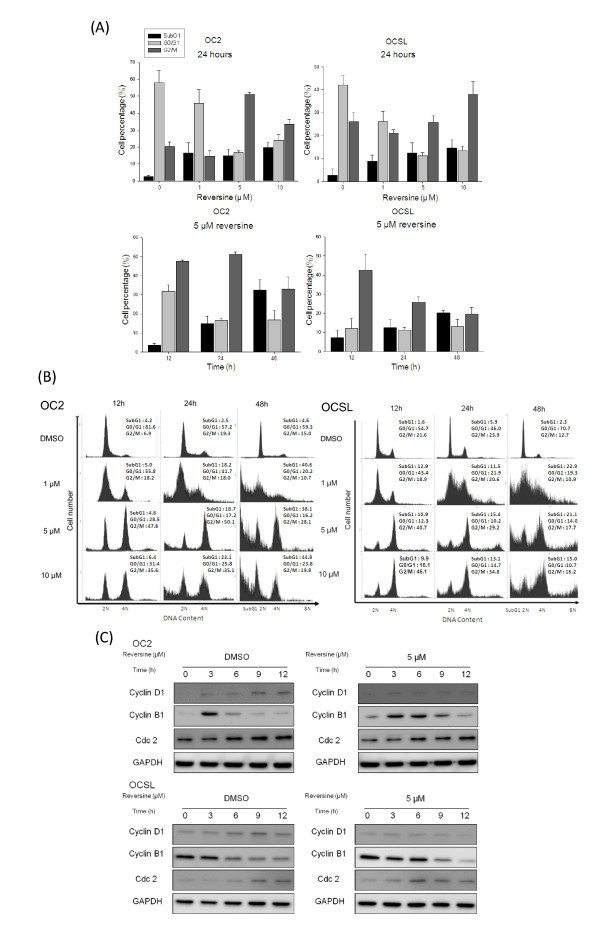
**Cell cycle arrests by reversine**. (A) OSCC cells were treated with DMSO or different concentrations of reversine (1, 5, 10 μM) for 12, 24 and 48 h. Cells were harvested, stained with PI and applied for flow cytometry analysis. The data were analyzed from three independent experiments. (B) As in (A), cells were treated with either different doses of reversine at 24 h or 5 μM reversine at indicated time points. The percentage of cells in each phase of the cell cycle was demonstrated. (C) OSCC cells treated with DMSO or 5 μM reversine were harvested and analyzed for cell cycle-dependent proteins. The GAPDH protein was used as the loading control.

Aurora kinases had been proved to play important roles in regulating cell division [34]. Inhibition of aurora kinases resulted in cell cycle arrest or even cell death [35,36]. To examine whether the cell cycle was affected, OC2 and OCSL cells were analyzed by flow cytometry after treating with various doses of reversine at different time points. The results were shown in Figure 2B. Basically, the percentage of G2/M cells increased with the high doses (10 μM) of reversine (Figure 2A upper panel). Taken In addition, the ratio of cells with 4N chromatin increased significantly in a dose- and time-dependent pattern (Figure 2B). Moreover, some cells with 8N chromatin were also noticed in OCSL cells, highly suggesting that reversine delayed progress of cell cycle and affected the processes of cytokinesis. To further support this notion, we checked the effect of reversine on the expression of cyclin B1. Reversine appeared to prolong the expression of cyclin B1 in synchronized OCSL and OC2 cells (Figure 2C). It is known that the expression of cyclin D1 is required for next cell cycle entry from M phase. Indeed, in the absence of reversine treatment (DMSO mock treatment), the levels of cyclin D1 proteins were up-regulated after 9 hours. In contrast, reversine treatment had suppressed the protein level of cyclin D1 throughout the treating period (Figure 2C). In addition, the levels of Cdc2, a CDK of G2/M, were not significantly influenced by reversine (Figure 2C). Taken together, these results indicated that reversine caused retardation at G2/M phase in the cell cycle. Moreover, reversine also highly increased subG1 population after 48 hours treatment (Figure 2A lower panel and 2B), suggesting a possibility that reversine induced cytotoxic effects at the later stage.

### Reversine induces cell death through canonical and non-canonical apoptosis pathways

**Figure 3:**
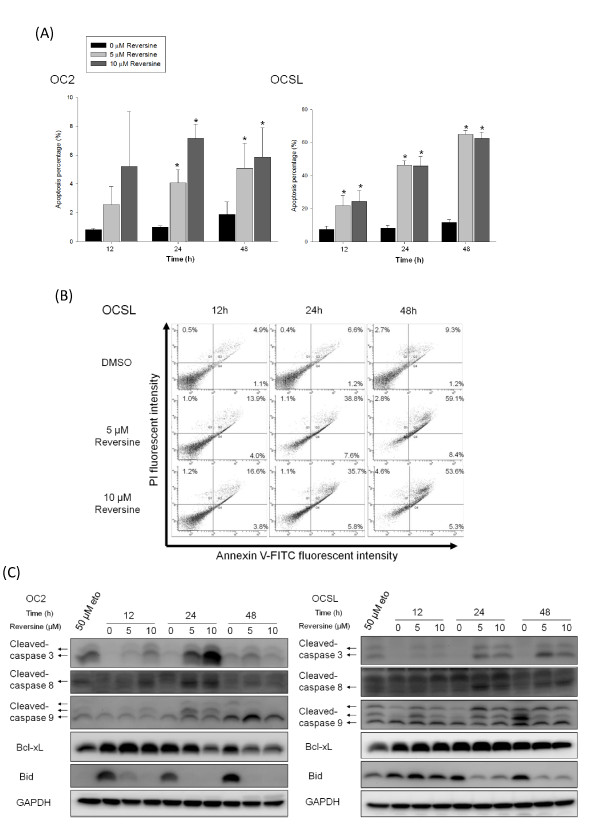
**Induction of apoptosis by reversine**. (A) OSCC cells were treated with DMSO or different concentrations of reversine (5 and 10 μM). At indicated time points (12, 24 and 48 h), cells were dually stained with annexin V-FITC and PI as described and analyzed by flow cytometry. The percentage of cells in early apoptosis and early apoptosis plus late apoptosis of OC2 and OCSL cells were evaluated, respectively. All results were obtained from three independent experiments. * indicated significant difference from the untreated cells (DMSO) (*p *< 0.05). (B) The representative figure of flow cytometry from OCSL cells treated as described in (A). (C) OSCC cells harvested from (A) were used to analyze the expression of apoptosis-related proteins. Cells treated with 50 μM etoposide to induce apoptosis were used as a positive control.

The increased subG1 population suggested growth suppression through programmed cell death. Therefore, OSCC cells were dually stained with annexin V-FITC and with propidium iodide (PI) to monitor type I programmed cell death (apoptosis) in the absence or presence of reversine. Treatment of reversine increased the population of annexin V positive and PI-positive cells in a time- and doses-dependent manner as shown in Figure 3A. Especially, the apoptosis percentage was over fifty in OCSL cells after 24 hours treatment. (Figure 3B), highly suggesting that reversine was more effective in OCSL cells, the cell line previously proven more malignant than OC2 [32].

Treatment of reversine obviously resulted in accumulation of cleaved caspase 3 (Figure 3C). This result further confirmed that reversine could suppress cell growth through type I programmed cell death. Both extrinsic and intrinsic pathways are involved in type I programmed cell death. We showed that the cleaved caspase 8 was increased after reversine treatment, indicating the activation of extrinsic pathway [37]. On the other hand, accumulation of processed caspase 9 and decrease of Bcl family members (Bcl-xL and Bid) demonstrated that reversine also enhanced intrinsic pathway. Taken together, these results confirmed that reversine could suppress the oral cell growth through the canonical caspase-dependent pathway.

**Figure 4:**
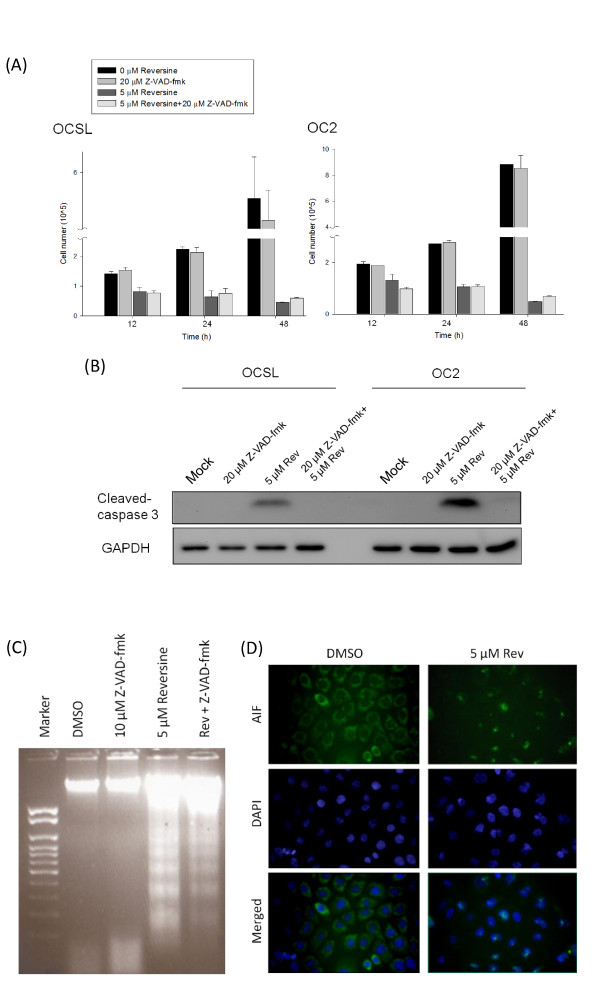
**Induction of caspase-independent cell death by reversine**. (A) Two hours before reversine addition, OSCC cells were pre-treated with or without 20 μM Z-VAD-fmk, followed by treated with or without 5 μM reversine, and harvested at 12, 24 and 48 h later. Cell numbers were counted as mentioned. (B) OSCC cells in (A) panel at 24th hour were used to monitor the cleaved caspase 3 protein as a marker of caspase-dependent apoptosis. (C) Similar to (B) panel, OC2 cells were used for DNA fragmentation analysis. (D) OC2 cells were treated with 5 μM reversine, stained with anti-AIF antibody and DAPI and visualized with a fluorescence microscope (magnification, ×200). AIF was retained in the cytoplasm in the absence of reversine and translocated into the nucleus after reversine treatment.

Z-VAD-fmk, a pan-caspase inhibitor, has been used to check whether drug-induced cell death is through the caspases pathway. Surprisingly, the number of viable cells only slightly increased even after treatment of inhibitor for two days (Figure 4A). It was unlikely due to insufficient concentration of Z-VAD-fmk inhibitor because of the complete inhibition of caspase 3 (Figure 4B). In addition, DNA ladder under the treatment of both Z-VAD-fmk and reversine was still observed (Figure 4C). Taken together, these results suggested that reversine-induced cell death was mediated mainly through another caspase-independent programmed cell death. To support these results, we examined the location of AIF in the absence or presence of reversine in OC2 cells. AIF was identified as a marker protein for caspase-independent apoptosis [38,39]. In normal physiological condition, AIF is retained in the mitochondrial membrane, where it executes the oxidoreductase function [40]. Once activated by apoptosis, AIF was translocated into the nucleus from the mitochondrial membrane and causes chromatin condensation and DNA fragmentation [41]. As shown in Figure 4D, AIF was located in the cytoplasm in the absence of reversine. However, after treatment of 5 μM reversine for 24 hour, AIF was translocated into the nucleus in OC2 cells, indicating that reversine can trigger the non-canonical caspase-independent cell death.

### Reversine also induces autophagy

**Figure 5:**
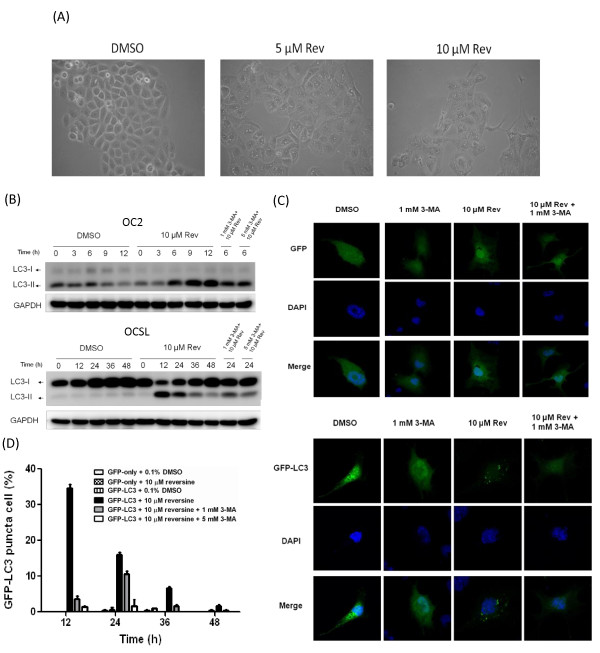
**Induction of autophagy by reversine**. (A) OC2 cells were treated without (DMSO) or with reversine (5 and 10 μM) for 24 h. Phase-contrast microscopy were taken with 200× magnification. Autophagic vesicles were observed in cells treated with reversine. (B) OSCC cells were pre-treated with 1 or 3 mM 3-MA for 2 hours, followed by treatment without or with 10 μM reversine. At indicated time points, cells were harvested for analyzing the cytoplasmic form LC3-I or the membrane form LC3-II. (C) OCSL cells were transfected with vectors expressing either GFP or GFP-LC3-II, followed by treatment of 3-MA and/or reversine as described. OSCL cells were analyzed under confocal microscope. (D) The same as (C) except that two different concentrations of 3-MA were evaluated. The percentages of cells with puncta were calculated by examining over 100 GFP positive cells. All values were expressed as means ± SD of three independent experiments, each of which was done in triplicate.

After treatment of reversine, cells showed intracellular vacuoles as shown in Figure 5A, implying that reversine may induce the autophagic responses. We checked whether reversine had an effect on the level of type I and II LC3 (LC3-II). LC3-II increased obviously within 12 hours after reversine treatment both in OC2 and OCSL cells (Figure 5B). Interestingly, the pattern of LC3 in OC2 and OCSL cells under normal condition was different. Even without reversine stimulation, OC2 cells showed endogenous LC3-II expression. Unlike in OC2, the effect of reversine on LC3-II in OCSL cells was more significant. Co-treatment of the autophagy inhibitor 3-MA decreased reversine-induced LC3-II in both cells, especially in OCSL cells (Figure 5B). This result indicated that reversine could trigger autophagy and might contribute to the caspase-independent programmed cell death. This possibility was further confirmed by the effect of reversine in LC3-II aggregation: In OCSL cells ectopically expressing GFP-LC3. GFP- LC3 showed diffuse pattern but became puncta pattern if treated with reversine (Figure 5C). This puncta pattern was not observed in the control cells harboring GFP plasmid only in the presence of reversine or 3-MA, ruling out the possibility of non-specific effects of the drugs on puncta formation. Moreover, this puncta pattern was totally abolished if co-treatment reversine with autophagy inhibitor 3-MA (Figure 5C). The quantitative results are shown in Figure 5D. These data confirmed that reversine triggered autophagic flux in OSCC cells.

### Reversine inhibits Akt/mTOR signaling pathway

**Figure 6:**
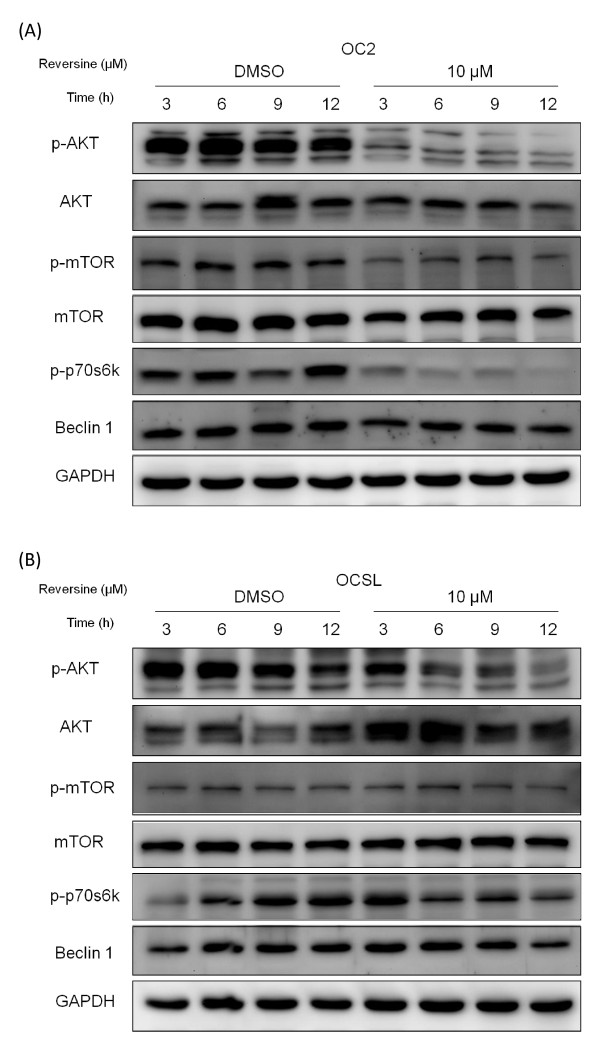
**Downregulation of Akt/mTORC1 signaling pathway by reversine**. OC2 cells (A) and OCSL cells (B) were treated with DMSO or 10 μM reversine and harvested at indicated time points for analyzing the indicated proteins, such as AKT, mTOR, p70S6K and Beclin.

Previous studies demonstrated that deregulation of PI3K-Akt signaling was frequently observed in various cancer cells including OSCC. For example, over-activation of Akt may enhance malignancy and resistance of cancer cells through anti- apoptotic stress [42]. Therefore, PI3K-Akt pathway became a promising target for cancer therapy recently [43]. In addition, aurora kinases were also proven as an upstream regulator of Akt activity in several cancers recently [44]. Since reversine was an inhibitor of aurora kinase, the effect of reversine on Akt activity deserved further investigations. As expected, reversine notably reduced the phosphorylation of Akt as well as downstream factors, including mTOR complex 1 (mTORC1) and p70S6K within 12 hours (Figure 6). On the contrary, although phospho-Jnk was affected slightly at later time, reversine showed no significant influence on the activation of MAPKs pathway (Additional file 2). These results indicated that reversine selectively down-regulated Akt/mTORC1 signaling pathway, resulting in the suppression of cancer cell growth and induction of autophagy.

## Discussion

Our previous studies have characterized two OSCC cell lines, OC2 and OCSL, which were established from buccal specimens of two Taiwanese male patients with habit of betel-quid chewing. The OCSL cells showed greater proliferation, horizontal and vertical migration, and transwell invasion abilities in comparison with OC2 cells [32]. In this study, we demonstrated that reversine suppressed the growth of these two OSCC cells. One of the mechanisms for such suppression is that reversine retards cell cycle at G2/M stage, which was evidenced by the prolonged expression of cyclin B1. This was also observed in the treatment of another aurora kinase inhibitor VX680 in HeLa cells [25,45]. However, we found that cyclin B1 decreased later in the treatment concurrently with an increased level of cyclin D (Figure 2C). This allows the cells to re-enter G1 phase, subsequently leading to 4N or even 8N chromosome content in OCSL cells (Figure 2B). Increase of polyploidy cells indicated the continuous DNA synthesis with unsuccessful cytokinesis. Consistent with this are growth arrest and polyploidy observed in HeLa, CWR22R*v*1, DU-145 and HCT-116 cells after reversine treatment [25,30,46].

We also demonstrated that reversine can trigger apoptosis, especially in the malignant OCSL cells (Figure 3A). The detail mechanism by which reversine triggers apoptosis remains unclear. However, we noticed that the amount of phosphorylated aurora kinases was slightly higher in OC2 than that in OCSL (Additional File 1, Figure S1). Previous study showed that aurora kinase A overexpression can override the mitotic spindle assembly checkpoint and induce resistance to taxol [47]. This study may explain why OC2 is more resistant to reversine. Moreover, VX680 selectively kills cells that overexpress c-myc. In other words, VX680 is more efficient to induce apoptosis in cells in a c-myc dependent but p53 independent manner [23]. In OSCC, p53 mutation and c-myc amplification were observed [48]. In OC2 and OCSL cells, both have mutated p53 but have the similar level of c-myc [49 and our unpublished date]. However, we did not rule out the possibility that these two OSCC cells potentially have mutated c-myc with different activity. Furthermore, inhibitor of aurora kinase B, ZM447439, suppresses the growth of cervical cancer SiHa cells and enhances the chemosensitivity to Cisplatin [50]. These studies provided the hint why OCSL was more sensitive to reversine.

Aurora kinases had been reported to participate in several signaling pathways like PI3K-Akt [44]. Here we show that reversine may inhibit the activity of Akt, which is frequently over-activated in many cancers [42,43]. Besides, mTORC1, the downstream factor of Akt, is also critical for cell proliferation and correlated to carcinogenesis [51]. Actually, mTORC1 phosphorylates 4E-BP1 to release eIF4E and affects the phosphorylation of ribosomal protein S6 through p70S6K [52]. Therefore, mTORC1 functions as a regulator for protein synthesis [53]. Interestingly, although reversine affects the activities of mTORC1 pathway, its final influence on translation machinery is not global based on the constant expression of Beclin 1 (Figure 6A). How the specificity was regulated still remains unclear.

In addition to proteins translation pathway, the role of mTORC1 is correlated to autophagy control [54]. Like double-edged sword, autophagy could have opposite effects on tumor cells [55]. Modest autophagy may help neoplasia cells survive under harsh environments [56,57]. Autophagy could play the protective role that impedes the cell death by reducing the occurrence of intrinsic apoptosis through mitochondria consumption [58,59]. On the contrary, autophagy was also reported to be disadvantageous for cancer cells [55-57]. Therefore, several autophagy-based chemicals are being tested for cancer therapy [60]. Our results showed that reversine enhances autophagy significantly in malignant OCSL cells, but weakly in less-malignant OC2 (Figure 5B). Figure 3A also suggested that reversine triggered apoptosis more effectively in OCSL cells. These discrepancies may be also suitable for differentiation between normal cells and cancer cells, which will be a tremendous advantage for the clinical application. Even under normal culture condition, we noticed that OC2 cells have high level of endogenous autophagy based on the constitutive expression of LC3-II (Figure 5B). Since OC2 cells is a less malignant cell line with the characteristics of squamous cells, it is highly possible that original OC2 cells may take advantage of high basal autophagy to survive before sufficient nutrients supply by angiogenesis in early carcinogenesis stage *in vivo*. Interestingly, inhibition of mTORC1 by rapamycin induces no significant increase of LC3-II in OCSL cells (data not shown), suggesting other unknown pathways involved in this reversine-induced autophagy in OCSL cells. The exact mechanism for reversine-induced autophagy in OCSL cells deserved to be verified.

Because several cancer cells were reported to have mutations such as p53 and caspases to enhance resistance against apoptotic stress, a multi-targeting strategy against tumor cells may increase the chance to treat cancers [61-65]. Here, we demonstrated that reversine is a broad-spectrum antitumor agent that induces cell cycle arrest, apoptosis, caspase-independent death and autophagy, suggesting that either reversine itself or reversine in combination with other drugs is a novel therapeutic regimen for OSCC patients.

## Conclusions

Oral cancer is one of the leading cancers in Taiwan due to betel quid chewing. However, current chemotherapy using Cisplatin and 5-Fu against OSCC remains inefficient to improve survival rate. Reversine suppresses OSCC cells via multiple mechanisms, which may provide a new way advantageous for treating oral cancer. Based on this study, evaluations of cellular sensitivity/resistance to reversine itself or reversine in combination with the current drugs Cisplatin and 5-Fu in cell culture and in animal xenografts model deserve to be further tested in the future.

## Competing interests

The authors declare that they have no competing interests.

## Authors' contributions

YRL initiated this project and paved the fundamental frame of this project. WCW executed most of the experiments. WTJ performed the Figure 4D, Figure 5A as well as additional file 1B and was responsible for writing the partial sections of the manuscript. JYFC conceived the plan. YPC and MKC finished the Figure 4C. HRC wrote the manuscript. All authors read and approved the final manuscript.

## Supplementary Material

Additional file 1 **Inhibition of aurora kinases activities by reversine**. (A) Aurora kinases were detectable in two OSCC cell lines, OC2 and OCSL. (B) Reversine inhibited Serine 10 phosphorylation of histone H3 in OSCC cells. Nocodazole treatment was used as a positive control. Click here for file

Additional file 2 **No significant influence on the activation of MAPKs pathway by reversine**. MAPK signaling pathway was not changed significantly in reversine-induced cell death. Proteins involved in this pathway, such as MAPK, Jnk and Erk were examined in OC2 and OCSL cells, respectively. The GAPDH protein was used as the loading control. Click here for file
