# Supplementary figures and images for: Reversine suppresses oral squamous cell carcinoma via cell cycle arrest and concomitantly apoptosis and autophagy
Source: J Biomed Sci. 2012 Jan 27;19(1):9. doi: 10.1186/1423-0127-19-9 (PMC3299600; doi:10.1186/1423-0127-19-9)

(A)

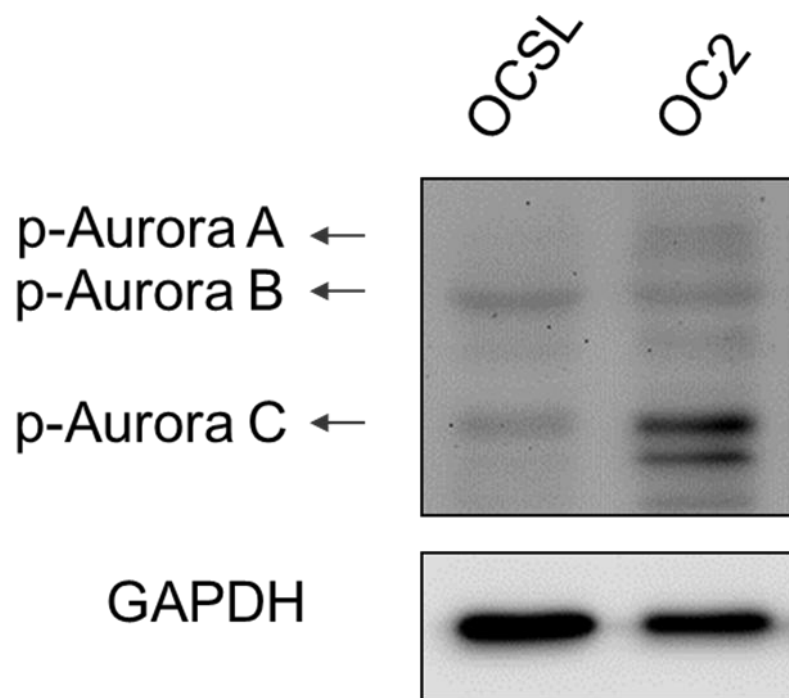

(B)

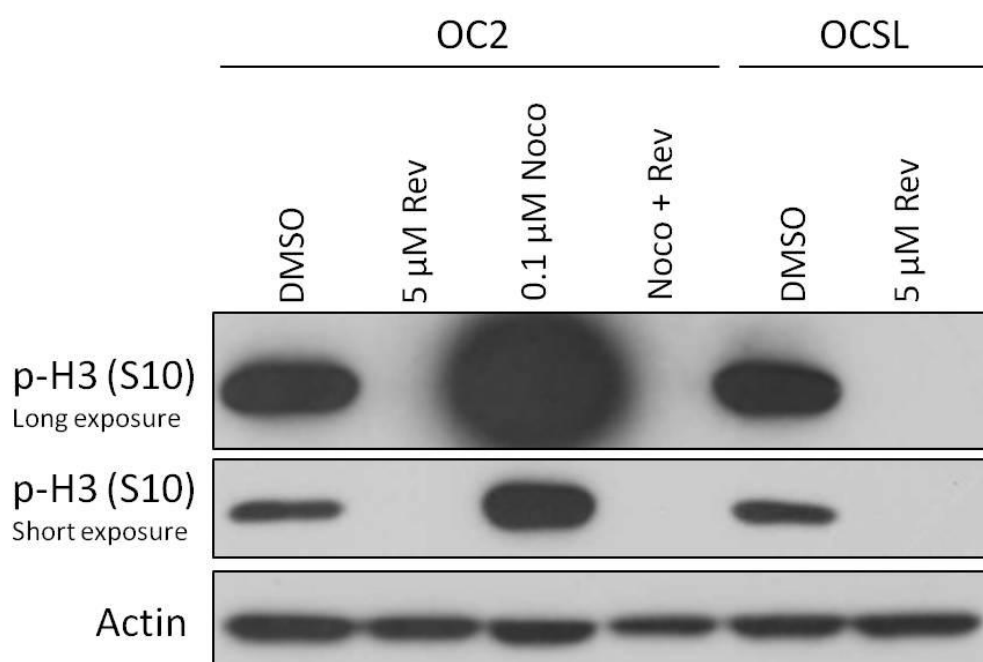

Supplement: Additional file 1 — Inhibition of aurora kinases activities by reversine. (A) Aurora kinases were detectable in two OSCC cell lines, OC2 and OCSL. (B) Reversine inhibited Serine 10 phosphorylation of histone H3 in OSCC cells. Nocodazole treatment was used as a positive control. [file 1423-0127-19-9-S1.PDF]

## Supplementary Figure 2

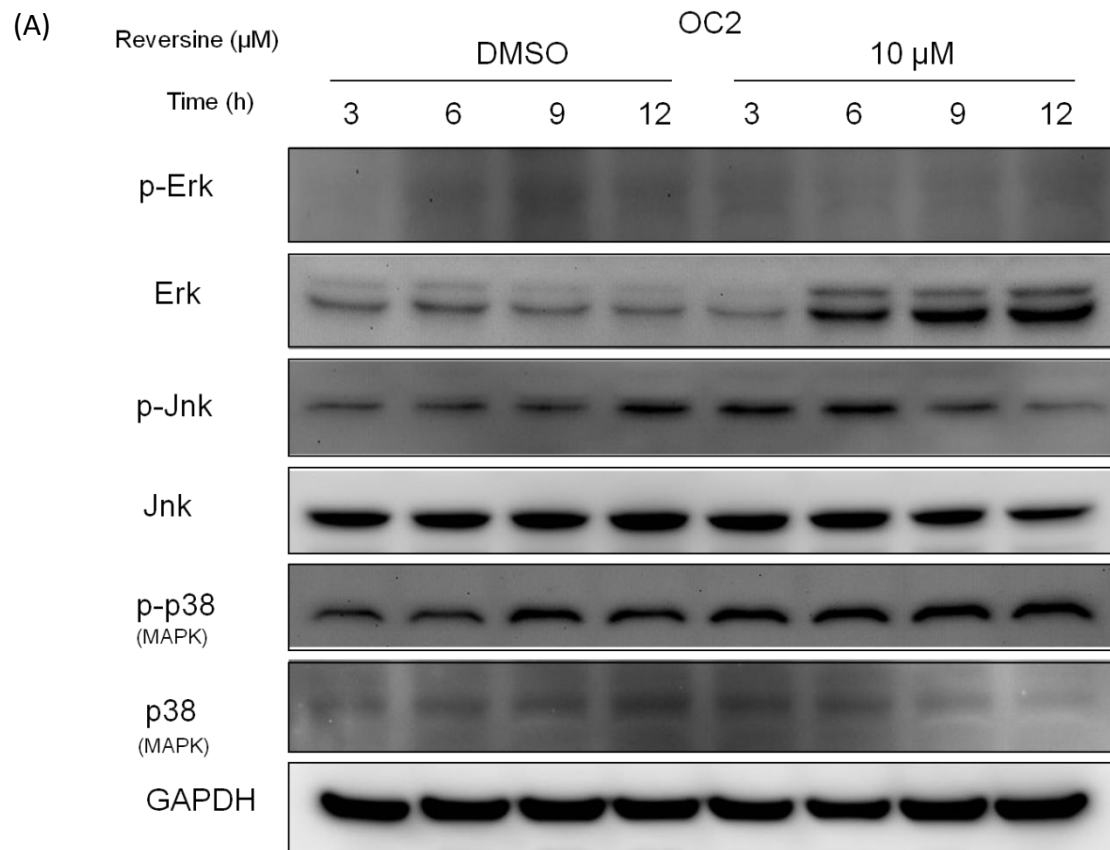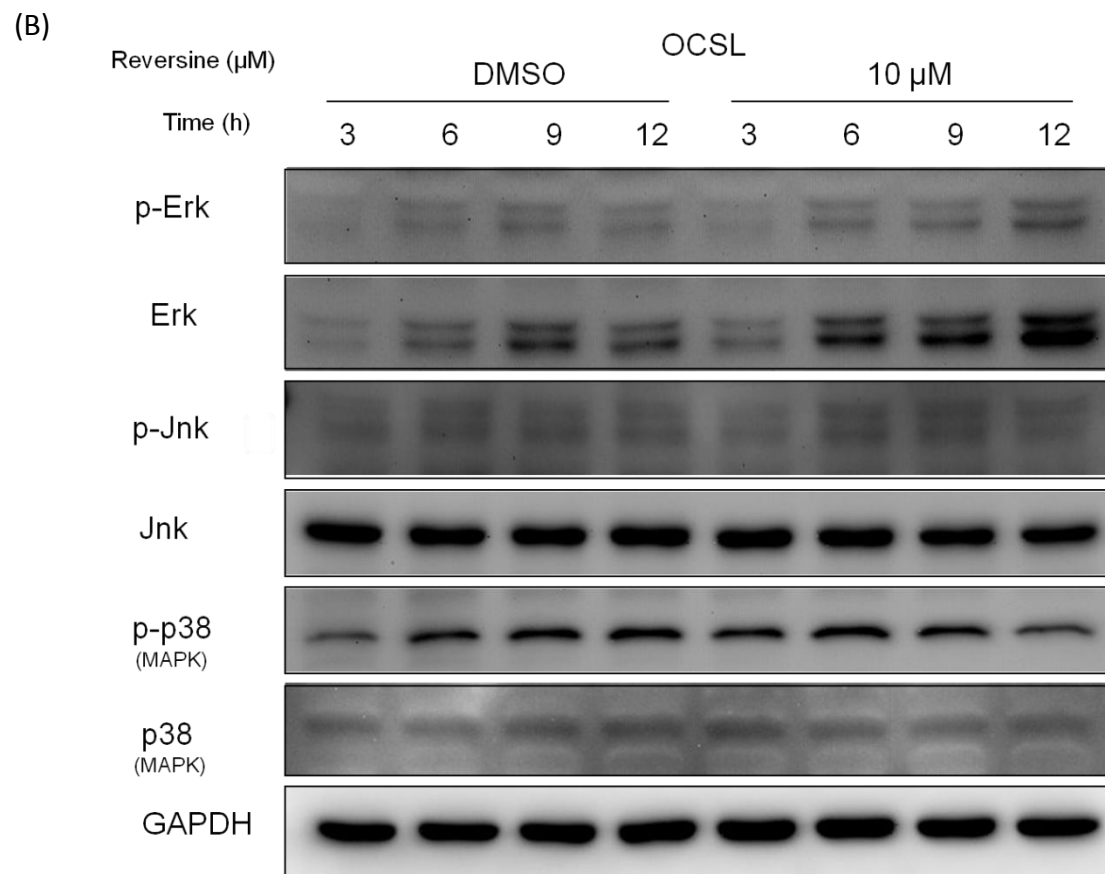

Supplement: Additional file 2 — No significant influence on the activation of MAPKs pathway by reversine. MAPK signaling pathway was not changed significantly in reversine-induced cell death. Proteins involved in this pathway, such as MAPK, Jnk and Erk were examined in OC2 and OCSL cells, respectively. The GAPDH protein was used as the loading control. [file 1423-0127-19-9-S2.PDF]
